# QM/MM Study of the Reaction Mechanism of L-Tyrosine Hydroxylation Catalyzed by the Enzyme CYP76AD1

**DOI:** 10.1021/acs.jpcb.4c05209

**Published:** 2024-08-26

**Authors:** João P. M. Sousa, Maria J. Ramos, Pedro A. Fernandes

**Affiliations:** LAQV-REQUIMTE, Departamento de Química e Bioquímica, Faculdade de Ciências Universidade do Porto, Rua do Campo Alegre, s/n, Porto 4169-007, Portugal

## Abstract

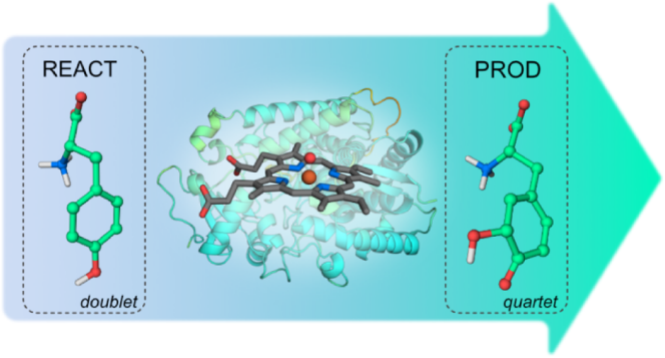

We have studied the hydroxylation mechanism of l-Tyr by the heme-dependent enzyme CYP76AD1 from the sugar beet (*Beta vulgaris*). This enzyme has a promising biotechnological application in modified yeast strains to produce medicinal alkaloids, an alternative to the traditional opium poppy harvest. A generative machine learning software based on AlphaFold was used to build the structure of CYP76AD1 since there are no structural data for this specific enzyme. After model validation, l-Tyr was docked in the active site of CYP76AD1 to assemble the reactive complex, whose catalytic distances remained stable throughout the 100 ns of MD simulation. Subsequent QM/MM calculations elucidated that l-Tyr hydroxylation occurs in two steps: hydrogen abstraction from l-Tyr by CpdI, forming an l-Tyr radical, and subsequent radical rebound, corresponding to a rate-limiting step of 16.0 kcal·mol^–1^. Our calculations suggest that the hydrogen abstraction step should occur in the doublet state, while the radical rebound should happen in the quartet state. The clarification of the reaction mechanism of CYP76AD1 provides insights into the rational optimization of the biosynthesis of alkaloids to eliminate the use of opium poppy.

## 1 Introduction

The diversity of plant secondary metabolism makes them an outstanding source of natural products with interesting biological properties. Benzylisoquinoline alkaloids (BIAs) are some of the plant secondary metabolites with the broadest application in traditional and classical medicine due to the analgesic properties of natural opioids. Morphine or codeine and the potent semisynthetic opioids, hydrocodone, or oxycodone stand out,^1,2^ giving them a spot on the WHO essential medicine list for the treatment of moderate and severe pain.^3^

A functional health system needs to maintain a steady supply of analgesic BIAs. The only economically viable method for obtaining these compounds is their extraction and isolation from *Papaver somniferum* (the opium poppy), which is alarming. Opium poppy farms are highly dependent on environmental conditions, which are evermore uncertain due to climate change. Uncertainty on the supply chain of these essential medicines puts increasing pressure on pharmaceutical companies to find viable alternatives for BIA production at an industrial scale.^4,5^

Microbial biosynthesis is one of the most promising alternatives for BIA production due to the easy manipulation and fast replication of organisms such as *Escherichia coli* and *Saccharomyces cerevisiae*.^6,7^

One of the most prominent developments has been synthesizing (*S*)-reticuline up to 4.6 g·L^–1^, a complex branch point intermediate in BIA biosynthesis, whose production was limited by available dopamine titers.^8−10^ Conversion of l-Tyr to dopamine is achieved through l-Tyr hydroxylation to L-DOPA and consecutive decarboxylation. Although the enzyme responsible for L-DOPA decarboxylation has been identified in the canonical BIA biosynthetic pathway, the canonical l-Tyr hydroxylase remains to be identified.^2,11^ Therefore, most microbial BIA biosynthesis applications have used other enzymes capable of catalyzing this conversion, such as the sugar beet CYP76AD^9,10,12^ or the mammalian pterin-dependent l-Tyr hydroxylase.^8^

CYPs catalyze a wide array of reactions in a wide variety of substrates, and their catalytic cycle has been extensively studied by both experimental and computational methods.

**Figure 1:**
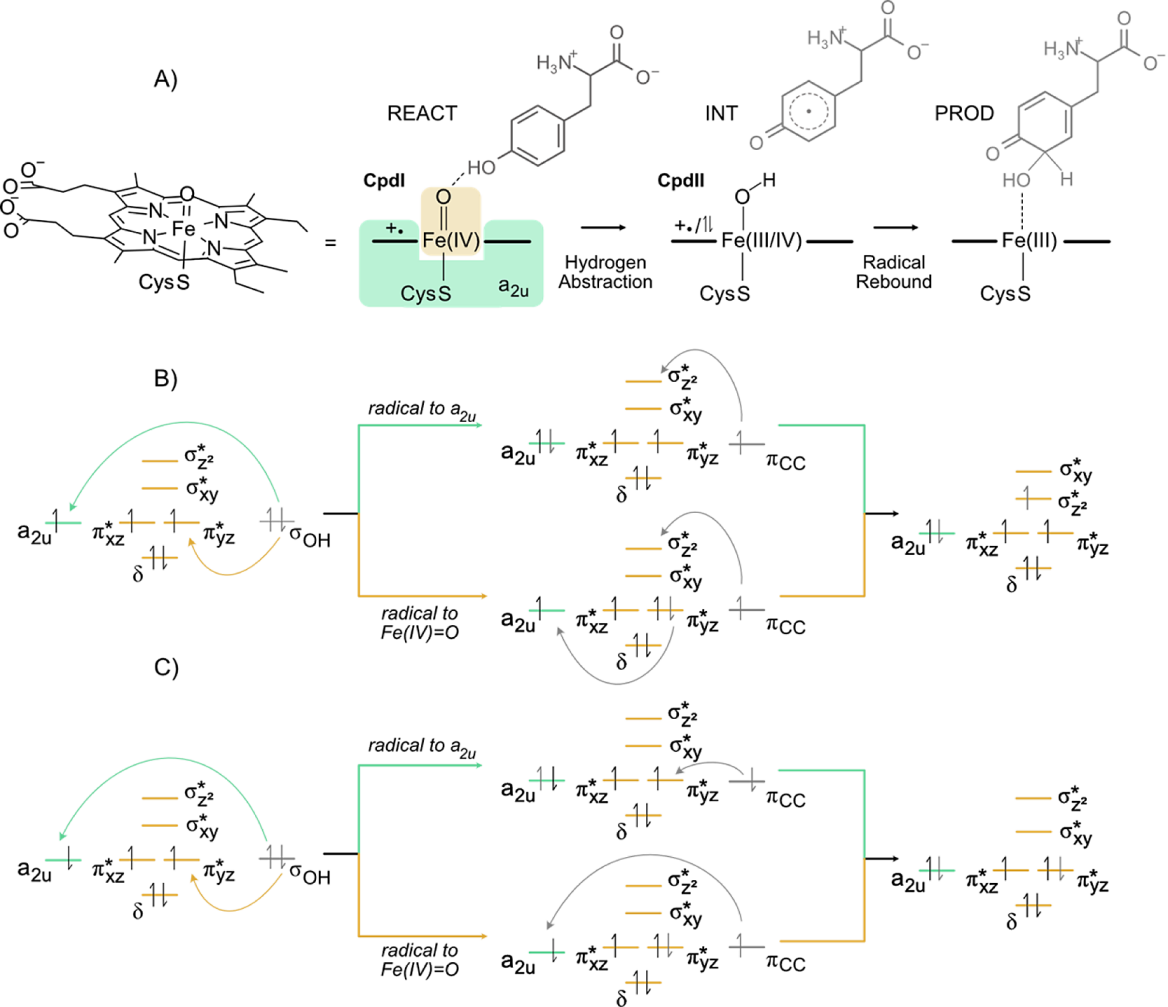
Mechanism proposal for l-Tyr hydroxylation. The mixed porphyrin-thiolate a_2u_ orbital is represented in green, and the Fe(IV)-oxo-based orbitals in light yellow; electron-transfer events during the l-Tyr hydroxylation by CpdI for the quartet and doublet spin states (B and C, respectively). In the first radical abstraction, radical pairing can occur either in the a_2_u MO or in the Fe(IV)-oxo MOs, therefore creating alternative pathways.

The hydroxylation of aromatic compounds by CYPs is carried out by compound I (CpdI)—the π-cation radical Fe(IV)-oxo species contained within the a_2u_ mixed porphyrin-thiolate molecular orbital (MO; Figure 1A).^13^

CpdI is a triradicaloid species that accommodates two unpaired electrons in the Fe(IV)-oxo-based MOs and another unpaired electron in the porphyrin-thiolate-based MO, a_2u_. The unpaired electron in the a_2u_ orbital is a result of a radical transfer from the porphyrin to the thiolate radical, which becomes a stronger electron acceptor due to its microenvironment within the enzyme, thus forming a cation radical in the a_2u_ MO and a thiolate anion.^13^ Depending on the spin nature of the radical abstracted by the thiolate, CpdI can be in a quartet spin state (Figure 1B) if the cation radical is in a “spin-up” orientation or a doublet spin state (Figure 1C) if the cation radical is in a “spin-down” orientation.

It was demonstrated in earlier studies that the homologous human CYP2D6 enzyme hydroxylates tyramine, the decarboxylated form of l-Tyr, through a mechanism where the cation radical from CpdI initially removes a hydrogen atom from the phenol moiety, forming a Fe(IV)-hydroxo complex (CpdII) and a phenoxy radical.^14−16^ In the second step, the radical rebounds to CpdII from the *ortho* position of the ring to form a bond between the carbon and the Fe(IV)-hydroxo ligand, thus forming keto-dopamine. The computed rate-limiting step of tyramine hydroxylation was radical rebound, accounting for an enthalpic barrier of 14.0 kcal.mol^–1^. Moreover, earlier studies elucidated that the keto-dopamine product will likely be converted into dopamine by a two-water assisted mechanism inside the active site or whenever the keto-dopamine diffuses to the solvent.^17^

We propose that the catalytic steps of the enzyme under study here, CYP76AD1, should be similar to those of CYP2D6 after CpdI formation, since they share a similar substrate and the same reactive cofactor (Figure 1A). In the present study, we will study the catalytic steps of l-Tyr hydroxylation by CYP76AD1 and trace its thermochemical profile through an electrostatic embedding QM/MM approach.

## 2 Methods

### 2.1 L-Tyr:CYP76AD1:CpdI Model Construction

#### 2.1.1 CYP76AD1:CpdI Model Prediction

CYP76AD1 has no available crystal structure; therefore, a model of the 3D structure of the enzyme was built with ColabFold 1.5.3^18−21^ using the sequence deposited under the UniProt entry I3PFJ5. In addition, a homology search was conducted with SwissModel^22−24^ to look for similar enzymes with available crystal structures, including a heme cofactor. From the set of templates generated from the SwissModel algorithm, the one with the best sequence identity (45%), corresponding to the substrate-free ferruginol synthase from *Salvia miltiorrhiza* (also known as CYP76AH1, following the typical CYP nomenclature) with the PDB code 5YLW, was selected to be superimposed with CYP76AD1 predicted with ColabFold 1.5.3 using VMD’s MultiSeq software.^25^ This procedure enabled the atomic coordinates of 5YLW to be fitted to the CYP76AD1 model and, subsequently, copy the coordinated protoporphyrin, Fe, and coordinating oxygen atomic coordinates from a crystallographic water molecule from the 5YLW structure to the CYP76AD1 model. The oxygen atom from the crystallographic water molecule was used to model the oxo ligand in the first coordination sphere of Fe.

#### 2.1.2 L-Tyr Docking in CYP76AD1:CpdI

The first 24 residues of CYP76AD1 correspond to a transmembrane helix structure, which we removed from the model, as they do not interfere with the substrate binding or catalytic events occurring in the active site. Force field parameters for CpdI with the axial thiolate cysteine ligand were retrieved from reference^26^ (provided in Supporting Information). Parameters for the zwitterionic l-Tyr substrate were created using the antechamber module of Amber18. The general Amber force field (GAFF)^27^ was used to generate the intramolecular and van der Waals parameters. Merz–Kollman charges were determined at the HF/6-31G(d) level. The GOLD docking algorithm^28^ generated binding poses for the l-Tyr substrate within 10 Å of the Fe(IV)-oxo ligand in the active site of previously minimized CYP76AD1:CpdI. The binding poses were ranked according to the CHEMPLP scoring function, and the complex with the best CHEMPLP score was selected for building the l-Tyr:CYP76AD1:CpdI reactive complex.^29^

### 2.2 MD Simulation of the L-Tyr:CYP76AD1:CpdI Complex

The protonation state of the ionizable residues of CYP76AD1 was determined at a pH of 7.0 using the ProteinPrepare web app.^30^ The LEaP module of Amber18^31^ was used to solvate the l-Tyr:CYP76AD1:CpdI complex in a cubic TIP3P water^32−34^ box whose edges were at least 12 Å away from the protein surface, resulting in the addition of 20950 TIP3P water molecules. The system charge was neutralized by adding 1 Cl^–^ counterion. Amber *ff*14SB^35^ was used to create parameters for protein atoms.

The assembled system was submitted to an energy minimization with the steepest descent algorithm to relax structural constraints arising from the described molecular modeling procedure. A harmonic force constant of 2000 kJ·mol^–1^·nm^–2^ was applied to all heavy atoms except for the solvent and the Cl^–^ counterion.

After the minimization stage, the system was equilibrated in the NVT ensemble for 100 ps and then for an additional 100 ps in the NPT ensemble, using the Berendsen barostat^36^ for pressure control. All heavy atoms were restrained with a harmonic force constant of 1000 kJ·mol^–1^·nm^–2^ and 500 kJ·mol^–1^·nm^–2^, for the NVT and NPT equilibrations, respectively.

Following the NPT equilibration, an unrestrained MD production stage was performed over 100 ns in the NPT ensemble, using the Parrinelo–Rahman barostat^37^ for pressure control at 1.0 bar using a time constant of 2.0 ps.

All MD stages used the velocity rescaling algorithm^38^ for temperature control at a target temperature of 290.15 K using a time constant of 0.1 ps. The LINCS algorithm was used to constrain the length of the bonds involving hydrogen atoms, allowing a time step of 2 fs to be integrated with the leapfrog algorithm for trajectory and atomic velocity prediction. The cutoff for the calculation of nonbonded interactions was set to 12 Å. vdW forces were smoothly turned off between 10 and 12 Å, and the calculation of long-range electrostatic interactions was performed by using the PME method. All energy minimizations and MD simulations were performed with the GROMACS 2021.5 software^39,40^

### 2.3 QM/MM Model Construction and Calculations

The QM/MM model was built from the minimized l-Tyr:CYP76AD1:CpdI structure using the ONIOM method.^41,42^ All water molecules and counterions were stripped from the model except for a water cap with a 5 Å thickness, which was kept to ensure the solvation of all residues at the enzyme surface.

For building the QM layer, the heme porphyrin was included together with the ferryl-oxo reactive species and the l-Tyr substrate side chain. The protein residues included in the QM model were Cys439, Pro440, Gly441, and Met442’s peptidic amino and Cα. In sum, the QM layer comprises 116 atoms (Figure S1) from a total of 11690 atoms. The QM layer has an overall charge of 0 atomic units, and both quartet and doublet low-lying spin states were considered for tracing the enzyme mechanism following the two-state reactivity typically found in CYP enzymes.^43−47^ Initially, a QM/MM geometry optimization was performed, where all atoms were allowed to minimize freely under the mechanical embedding scheme. After that, all atoms further than 15 Å from the QM layer were fixed, and a QM/MM geometry optimization under the electrostatic embedding scheme was performed.

Linear transit scans were performed along the tentative reaction coordinates. The maximum energy structures were isolated and submitted to TS geometry optimization. Vibrational frequency calculations were performed in the optimized TS structures to confirm the existence of a single imaginary frequency corresponding to the bond-breaking/formation events. IRC calculations in the forward and reverse directions traced the path connecting the TS to the correspondent minima. Once the minimum structures from the IRC calculations were obtained, they were submitted to QM/MM geometry optimization under the electrostatic embedding scheme.

All QM/MM geometry optimizations, vibrational frequencies, and IRC calculations employed the B3LYP/6-31G(d):*ff*14SB level of theory, and the single-point energy calculations on each stationary point employed the B3LYP/6-311+G(2d,2p)-D3:*ff*14SB level of theory with Grimme’s D3 dispersion and Becke–Johnson damping corrections (stationary point geometries and absolute energy values are provided in Supporting Information).^48−52^ The choice of DFT with the B3LYP functional was based on the previous comparisons with both experimental and high-level theoretical methods using several CYP enzymes, which selected this method as the one with the best cost/accuracy.^13,53,54^ Zero-point, thermal, and entropic (rigid rotor/harmonic oscillator) corrections were added to the free energy. The atomic charges and spin densities of the QM layer atoms of each stationary point were calculated using the Hirshfeld charge method.^55−57^ The atoms considered for the calculation of the charge and spin density of the a_2u_ orbital are all porphyrin heavy atoms and Cys439 sulfur atoms (see Figure S1).

Gaussian 16 revision B.01^58^ was used to conduct all electronic structure calculations.

## 3 Results and Discussion

### 3.1 Evaluation of the L-Tyr:CYP76AD1:CpdI Model

**Figure 2:**
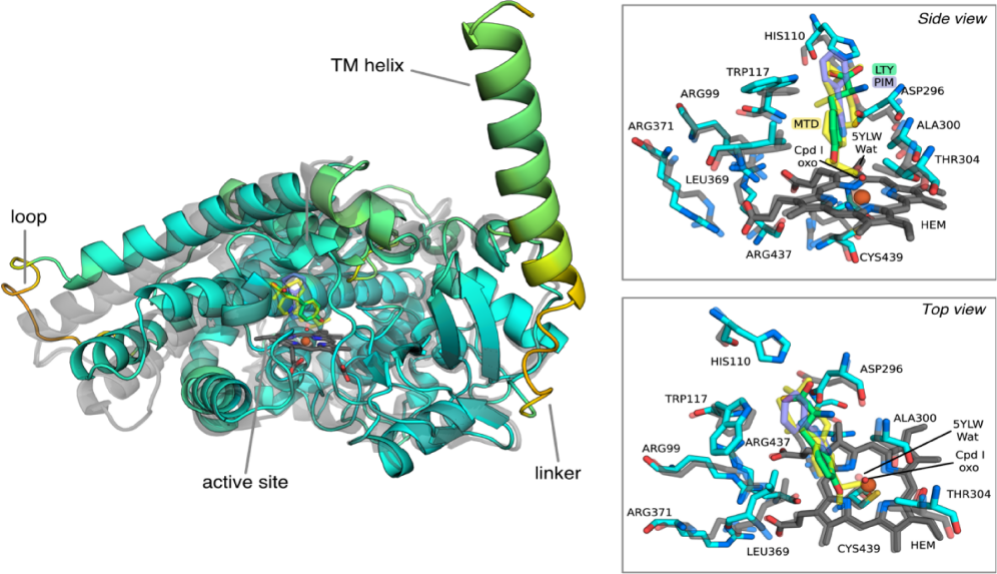
Alignment of the predicted model with the 5YLW, 5YM3, and 7CB9 X-ray structures. A color spectrum from orange to cyan is shown as a visual indicator of the pLDDT score: low pLDDT regions are colored in orange, and high pLDDT regions are colored in cyan. The X-ray structures are shown as transparent representations. The panels show a side and top view of the active site and the predicted pose of the l-Tyr substrate relative to PIM (5YM3) and MTD (7CB9).

The predicted model had 474 of 497 residues with a pLDDT score above 70%, indicating a high-confidence model. The regions with a pLDDT score below 70% correspond to a linker to the TM helix (residues 21 to 30) and to a solvent-exposed loop (residues 259–269), which are distant from the active site and do not play a role in catalysis (Figure 2). The alignment of the predicted model with the X-ray structure of the substrate-free ferruginol synthase (PDB accession code: 5YLW; shown in dark transparency in Figure 2) shows that both structures are highly similar except for the loop region with a low pLDDT score. Furthermore, the active site of both enzymes is highly conserved with an active site RMSd of 1.27 Å and an active site identity of 82%, only differing in residues Trp117 and Ala300, which in ferruginol synthase correspond to Phe113 and Gly298, respectively.

The docking best-ranked pose shows that l-Tyr is lodged in a cavity in the distal face of the heme. As there are no available crystal structures of CYP76AD1, the docking validation was performed by comparing the predicted binding pose for l-Tyr with available crystal structures of ferruginol synthase from *Salvia miltiorrhiza* with bound inhibitors 4-phenylimidazole (PIM; PDB accession code: 5YM3) and miltiradiene (MTD; PDB accession code: 7CB9).

As CYP76AD1 and ferruginol synthase share the typical CYP fold, 45% of their whole sequence, and also share most of the active site residues, the substrate binding site location should also be similar. Indeed, the alignment of CYP76AD1 with the PIM and MTD-bound ferruginol synthase confirmed that the location of both ferruginol synthase inhibitors matches that of the predicted l-Tyr binding pose in the CYP76AD1 active site (Figure 2). A closer inspection of the binding pose reveals that His110 and Trp117 amino groups are within 1.7 Å of the l-Tyr carboxylate group. The Asp296 carboxylate is also within 1.9 Å of the l-Tyr positively charged amino group. The l-Tyr phenol interacts with the oxo ligand through a 2.1 Å hydrogen bond between the hydroxyl and the Fe(IV)-oxo ligand (Figure S2).

### 3.2 MD Simulation of the L-Tyr:CpdI:CYP76AD1 Complex

The RMSd analysis of the protein backbone reveals an increase to about 2.3 Å in the first 2 ns of the production stage, eventually decreasing to about 1.3 Å but increasing gradually along the simulation.

Visual analysis of the 100 ns MD reveals that the N-terminus and the solvent-exposed loop (Figure 2) are highly flexible and most likely cause the RMSd increase. A backbone RMSd analysis excluding the N-terminal backbone (residues 25–31) and the solvent-exposed loop (residues 258–271) results in the absence of the abrupt increase in the beginning of the production stage and the stabilization of the backbone around 1.1 Å after 45 ns (Figure S3).

**Figure 3:**
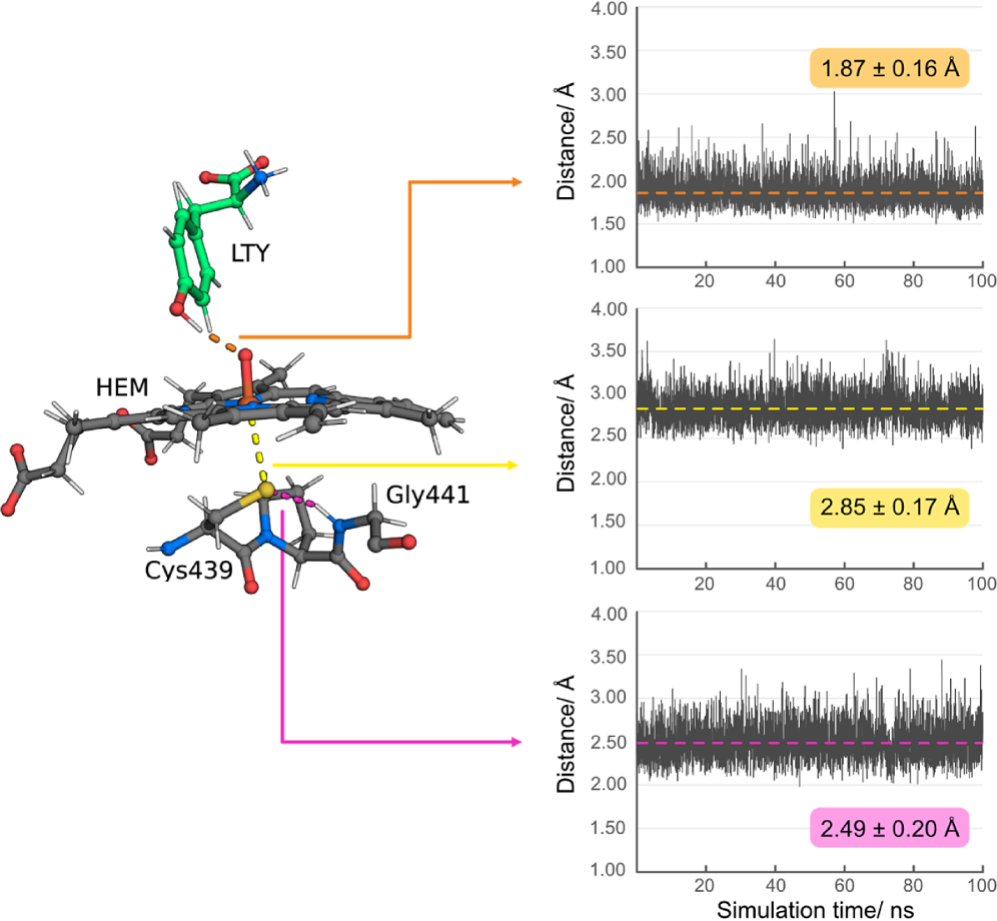
Catalytic distance analysis of the 100 ns CYP76AD1 trajectory: distance of the hydrogen bond interaction between the l-Tyr hydroxyl group and CpdI oxo group (orange). Coordination distance between the Fe(iV) center and Cys439 thiolate group (yellow). Distance of the hydrogen bond interaction between the Cys439 thiolate group and Gly441 peptidic amino group (pink).

The l-Tyr substrate RMSd oscillates between 0.3 and 1.7 Å (Figure S3). Nevertheless, the interaction between the l-Tyr hydroxyl and the oxo ligand remains prevalent throughout the simulation (1.87 ± 0.16 Å; Figure 3). A closer inspection of the l-Tyr trajectory indicates that the oscillation in RMSd might be due to rotation of the carboxylate group about the Cα-CO_2_ bond axis. As for the CpdI cofactor, its RMSd stabilizes around 0.6 Å after 48 ns. The Cys439 thiolate ligand remains coordinated to the iron center throughout the entire MD (2.85 ± 0.17 Å) and is stabilized by the peptidic amino group of Gly441 (2.49 ± 0.20 Å; Figure 3).

The results from the MD production stage suggest that the enzyme–substrate complex equilibrates after 45 ns of simulation and that the crucial interactions for the subsequent study of the reaction mechanism are stable throughout the 100 ns MD simulation.

### 3.3 L-Tyr Hydroxylation Mechanism

#### 3.3.1 Step1: Hydrogen Abstraction

**Figure 4:**
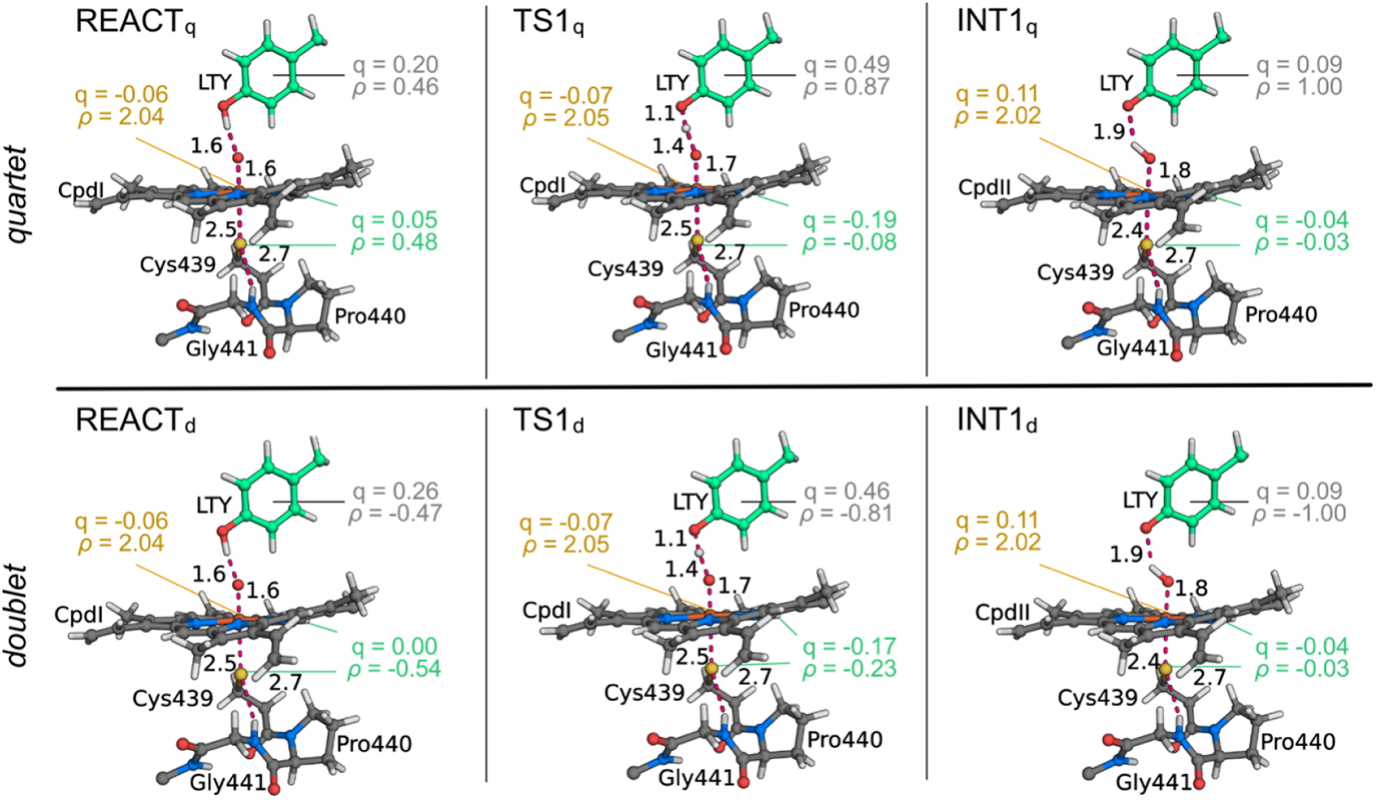
Geometry of the QM layer at the stationary points of the hydrogen abstraction step. Hirshfeld partial charges (q) and spin densities (ρ) for l-Tyr (LTY), a_2u_, and Fe(IV)-oxo are colored gray, green, and light yellow, respectively. Distances are shown in angström.

Our calculations show that the reactant state in the doublet (REACT_d_) is stabilized at 3.3 kcal·mol^–1^ relative to the quartet (REACT_q_). The REACT geometry is characterized by a “side-on” approach of l-Tyr’s phenol relative to the porphyrin plane (Figure 4), establishing a hydrogen bond with the Fe(IV) oxo ligand. The Fe···S distance is 2.5 Å and the Fe···O is 1.6 Å which are consistent with experimental X-ray spectroscopy values determined by Stone and co-workers (Fe···S = 2.48 Å and Fe···O = 1.65 Å) and with DFT/MM studies performed by Bathelt and co-workers.^59^

A spin density (ρ) of 2.04 is found in the Fe(IV)-oxo-based MOs, due to the two unpaired electrons in the π* orbitals. In both REACT_d_ and REACT_q_, the cation radical is delocalized between the thiolate (^doublet^ρ_S_ = −0.19; ^quartet^ρ_S_ = 0.21), the porphyrin (^doublet^ρ_porph_ = −0.35; ^quartet^ρ_porph_ = 0.27), and the substrate orbitals (^doublet^ρ_LTY_ = −0.47; ^quartet^ρ_LTY_ = 0.46). The calculated spin densities in both reactant states for the thiolate are in agreement with the electronic nuclear double-resonance spectra of the rapid freeze quenched CpdI state of chloroperoxidase which demonstrated a spin density of approximately 0.23 in the thiolate.^60^ Previous DFT/MM studies also report a variation of 26% to 50% in the spin density of the thiolate ligand, according to the model setup.^59^ In CYP76AD1, we found that the electron donor character of the l-Tyr substrate enables the delocalization of the radical to the substrate orbitals in the reactant state. This electron donor effect of l-Tyr explains its high-spin density and is similar to what has been observed in other CYPs and peroxidases whose substrate is an electron donor and the cation radical is spread over the thiolate, the porphyrin, and the substrate.^61^ The REACT stationary states are closer to the respective TS1 than to a structure where l-Tyr does not establish the hydrogen bond with CpdI, which is suggested by the accumulation of spin density in the l-Tyr substrate, even before hydrogen abstraction and the doublet-to-quartet energy gap.

Our calculations indicate that l-Tyr hydrogen abstraction and complete radical delocalization to the substrate are almost barrierless in both TS 1d (*i* = 639.1 cm^–1^) and TS 1q (*i* = 398.1 cm^–1^), with barriers of 0.7 and 1.7 kcal/mol, respectively (Figure 6). Furthermore, the formation of the CpdII:l-Tyr cation radical (l-Tyr^•+^) intermediate (INT1) complex is thermodynamically favored, with a Gibbs reaction free energy of −9.8 kcal·mol^–1^ relative to REACT_d_.

INT1_d_ is about 1.4 kcal·mol^–1^ more stable than INT1_q;_ nevertheless, a slight tilt of the phenoxy moiety leads to degenerate INT2, in which both states are separated by less than 0.5 kcal·mol^–1^. INT2 was detected as a result of a reverse IRC calculation starting from the transition state of the subsequent reaction, TS2, whereas INT1 resulted from a forward IRC calculation starting from TS1.

#### 3.3.2 Step 2: Radical Rebound

**Figure 5:**
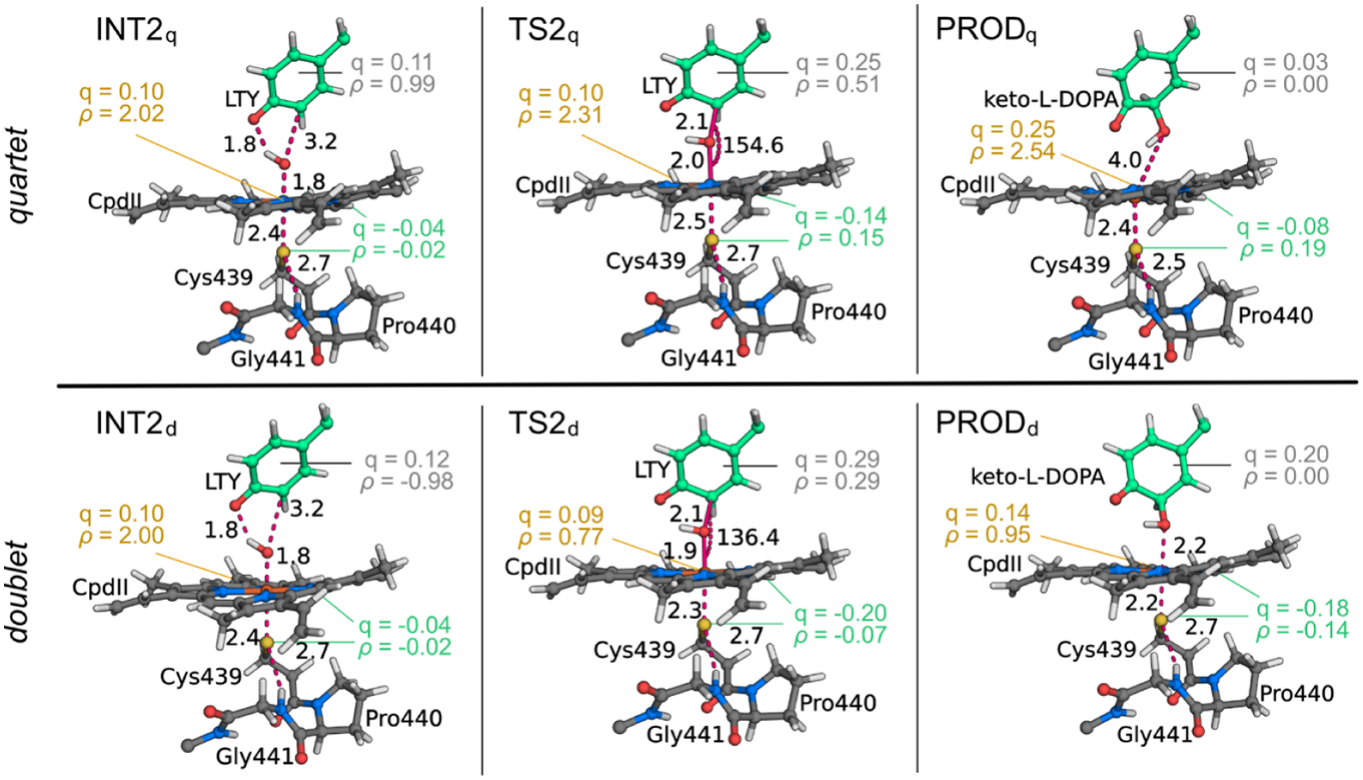
Geometry of the QM layer in the stationary points of the radical rebound step. Hirshfeld partial charges and spin distributions for l-Tyr (LTY), a_2u_, and Fe(IV)-oxo are represented in gray, green, and light yellow, respectively. Distances are shown in angström and angles in degrees.

**Figure 6:**
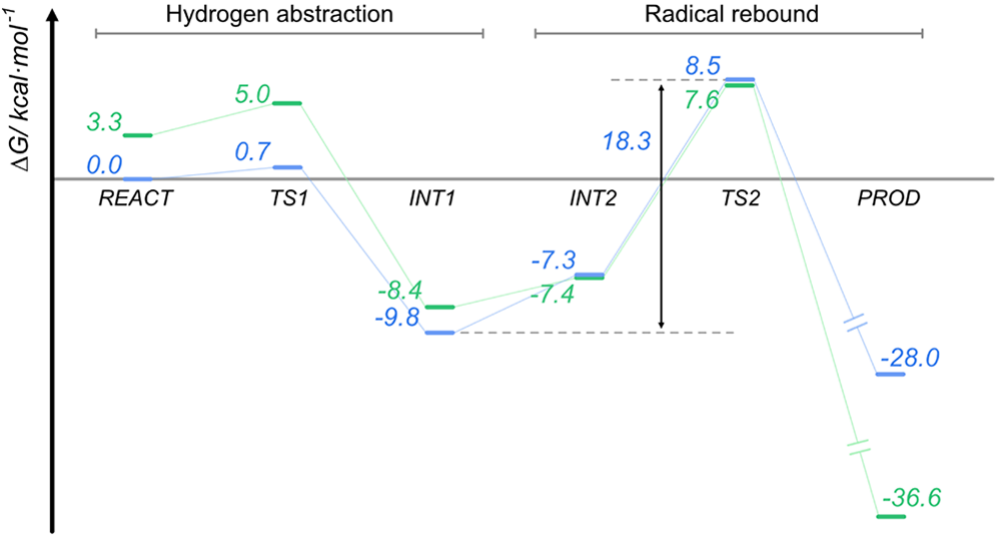
Gibbs free-energy profile for the quartet (green) and doublet (blue) spin states. The dashed lines delimit the rate-limiting step and the respective Gibbs free energy of activation.

Spin density distribution in INT1 and INT2 suggests that CpdII is characterized by a closed-shell a_2u_ and a Fe(IV)-hydroxo coordination complex in both spin states (Figure 1B,C). At the same time, the cation radical is fully formed on the phenoxy moiety of the substrate, with a “spin-up” (positive ρ) configuration in the quartet and a “spin-down” (negative ρ) in the doublet.

l-Tyr^•+^ radical rebound to CpdII occurs with a Gibbs free energy of activation (Δ*G*^‡^) of 15.9 kcal·mol^–1^ relative to INT2_d_ and 14.8 kcal·mol^–1^ relative to INT2_q_, making the conversion of INT1 to TS2 the rate-limiting step of the CYP76AD1 mechanism (Figure 6). A comparison of TS2_d_ (*i* = 608.2 cm^–1^) and TS2_q_ (*i* = 530.0 cm^–1^) shows that the tilt in the phenoxy moiety relative to the porphyrin is more pronounced in TS2_q_, which leads to a more linear Fe–O–C angle in TS2_q_ (155°) than in TS2_d_ (136°). Also, Fe···OH and Fe···S are longer in TS2_q_ than in TS2_d_. It is also worth mentioning that the total spin density is conserved, with the unpaired electrons mainly delocalized across both iron-oxo and l-Tyr^•+^ moieties. Note that the spin density in the doublet state increases from −0.98 to 0.29, and this increase should not be due to a spin-crossing event but rather to the delocalization of the single electron in the Fe-oxo-based orbitals. This is further supported by the reduction in spin density in Fe-oxo (Figure 5).

In PROD_q_, keto-L-DOPA readily dissociates from the Fe(III) center. In contrast, in PROD_d_, the keto-L-DOPA remains coordinated to the metal, leaving hexa-coordianted Fe(III). The reaction is highly exergonic in both cases—–36.6 kcal·mol^–1^ for PROD_q_ and −28.0 kcal·mol^–1^ for PROD_d_, meaning PROD_q_ is stabilized in about −8.6 kcal·mol^–1^ relative to PROD_d_.

The full free-energy profile is shown in Figure 6, showing that the rate-limiting step corresponds to 18.3 kcal·mol^–1^ and 16.0 kcal·mol^–1^ for the reaction in the doublet state and the quartet state, respectively. Therefore, the reaction in the quartet state is thermodynamically and kinetically favored.

In either case, entry of water molecules into the active site should reestablish the resting state of the enzyme, in which a water molecule will coordinate Fe(III) in the distal axial position, and conversion of keto-L-DOPA into L-DOPA should occur upon release of keto-L-DOPA into solution, in a two-water-mediated and enzyme-independent mechanism, as predicted by Schyman and co-workers.^17^

## 4 Conclusion

In this study, we have dissected the CYP76AD1 reaction mechanism of l-Tyr hydroxylation by Fe(IV)-oxo species with a cation radical in the a_2u_ MO, commonly known as CpdI.

The first step of the reaction is synchronous electron-transfer and proton abstraction from l-Tyr, forming the CpdII:l-Tyr•+ reactive complex. CpdII is characterized by a closed-shell a_2u_ and aFe(IV)-hydroxo, which will hydroxylate l-Tyr^•+^, forming the keto-L-DOPA product in the second and rate-limiting step.

The thermochemical profile was calculated for the low-energy doublet and quartet spin states. In both spin states, the rate-limiting step is the l-Tyr^•+^ radical rebound step with a Δ*G*^‡^ of 18.3 kcal·mol^–1^ for the doublet and 16.0 kcal·mol^–1^ for the quartet, which is in line with results from previous DFT/MM studies.^17^ The QM layer geometry, charge, and spin distribution of the stationary points also agree with DFT/MM and experimental studies.^60,62,63^

The identification and characterization of the stationary point geometry, charge, and spin distribution shed light on the l-Tyr hydroxylation mechanism by the CYP76AD1 enzyme. The findings of this study can be employed as a basis for the development of enhanced CYP76AD1 variants with the objective of augmenting BIA biosynthesis in an opium-poppy-free manner, using heterologous organisms.

## Supporting Information Available

Figure detailing the atoms included in the QM layer and the atoms considered for the a_2u_ orbital (Figure S1); table with the distribution of the spin density and Hirshfeld partial charges in the QM layer for all stationary points of both quartet and doublet spin states (Table S1 and Table S2); figure of the l-Tyr docking pose interactions with CYP76AD1 (Figure S2); RMSd graphs detailing the stability of the heme, the l-Tyr substrate, and CYP76AD1 backbone (Figure S3); a table showing the enthalpic contribution, the correction to Gibbs free energy at 298.15K, and the final Gibbs free-energy values for all stationary states (Table S4); Amber compatible force field parameters used for CpdI, the atomic charges for Cys439, and the atomic charges for the heme cofactor and oxo ligand (Pages S4–S9) (PDF)
Stationary point structures and associated absolute single-point energy values (XYZ)

## Supplementary Material

jp4c05209_si_001.pdf

jp4c05209_si_002.xyz
