# Supplementary material for: QM/MM Study of the Reaction Mechanism of L-Tyrosine Hydroxylation Catalyzed by the Enzyme CYP76AD1
Source: J Phys Chem B. 2024 Aug 26;128(39):9447–54. doi: 10.1021/acs.jpcb.4c05209 (PMC11457145; doi:10.1021/acs.jpcb.4c05209)
Supplement: Supplementary file 1 — jp4c05209_si_001.pdf [file jp4c05209_si_001.pdf]

Supplementary Information

QM/MM Study of the Reaction Mechanism of L-Tyrosine Hydroxylation Catalyzed by the Enzyme CYP76AD1

João P. M. Sousa[a], Maria J. Ramos[a], Pedro A. Fernandes[a]*

[a]LAQV-REQUIMTE, Departamento de Química e Bioquímica, Faculdade de Ciências

Universidade do Porto, Rua do Campo Alegre, s/n, 4169-007 Porto, Portugal

*E-mail: pafernan@fc.up.pt

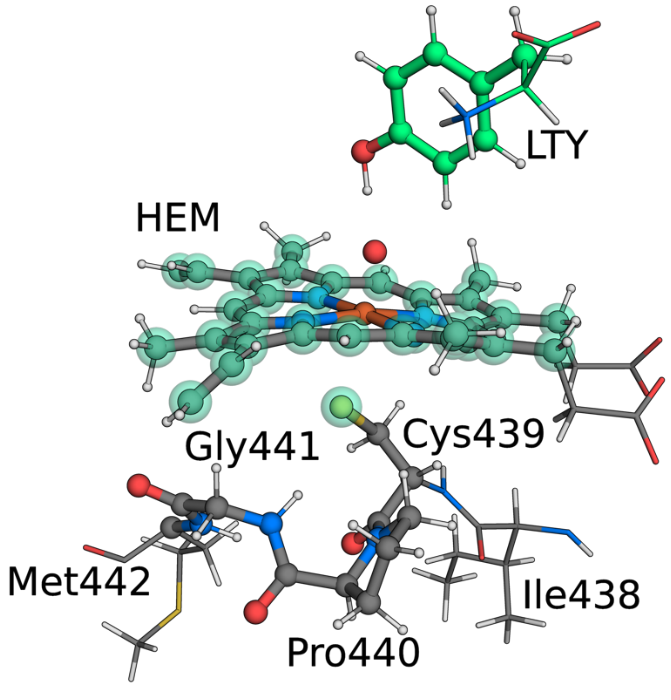

Figure S1- QM layer of CYP76AD1 QM/MM model. Thicker spheres correspond to the atoms included in the QM layer. Thiner sticks correspond to truncated MM layer atoms. The a_{2u} mixed porphyrin-thiolate orbital atoms are highlighted with green transparent spheres.

Table S1 – Hirshfeld charged (q) and spin densities (ρ) for the Fe ligands and substrate for all stationary points of the quartet spin state reaction.

		REACTq	TS1q	INT1q	INT2q	TS2q	PRODq
Cys439 thiolate	q	-0.14	-0.21	-0.14	-0.15	-0.20	-0.19
	ρ	0.21	0.01	0.02	0.03	0.15	0.27
Porphyrin	q	0.19	0.02	0.10	0.10	0.06	0.11
	ρ	0.27	0.07	-0.05	-0.05	0.00	0.13
Fe	q	0.20	0.20	0.25	0.24	0.25	0.25
	ρ	1.41	1.50	1.76	1.75	2.39	2.54
Oxo	q	-0.26	-0.28	-0.13	-0.15	-0.15	-0.12
	ρ	0.63	0.55	0.26	0.27	-0.08	0.00
LTY	q	0.20	0.49	0.09	0.11	0.25	0.15
	ρ	0.46	0.87	1.00	0.99	0.51	0.00

Table S2 - Hirshfeld charged (q) and spin densities (ρ) for the Fe ligands and substrate for all stationary points of the doublet spin state reaction.

		REACTd	TS1d	INT1d	INT2d	TS2d	PRODd
Cys439 thiolate	q	-0.17	-0.19	-0.14	-0.15	-0.16	-0.11
	ρ	-0.19	-0.05	0.02	0.03	0.03	0.05
Porphyrin	q	0.17	0.03	0.10	0.10	-0.05	-0.07
	ρ	-0.35	-0.19	-0.05	-0.05	-0.09	-0.01
Fe	q	0.20	0.20	0.25	0.24	0.25	0.14
	ρ	1.41	1.50	1.76	1.75	2.39	0.95
Oxo	q	-0.26	-0.27	-0.13	-0.15	-0.10	-0.03
	ρ	0.62	0.53	0.25	0.25	-0.13	0.01
LTY	q	0.26	0.46	0.09	0.12	0.29	0.23
	ρ	-0.47	-0.81	-1.00	-0.98	0.29	0.00

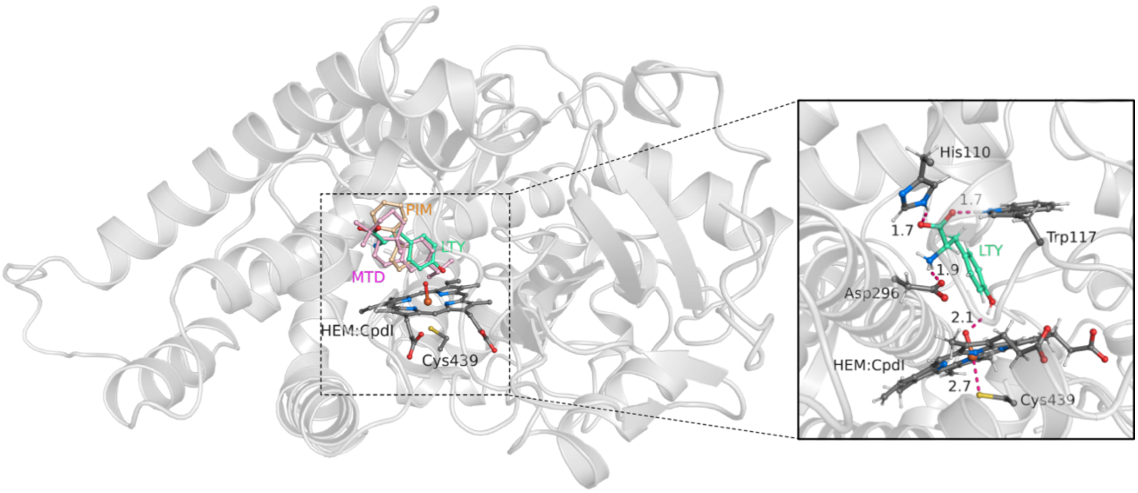

Figure S2- L-Tyr docking in CYP76AD1 and its alignment with the ferruginol synthase inhibitors PIM (PDB accession code: 5YME) and MTD (PDB accession code: 7CB9). The left panel shows interactions between the L-Tyr substrate and CYP76AD1 residues found in L-Tyr best pose.

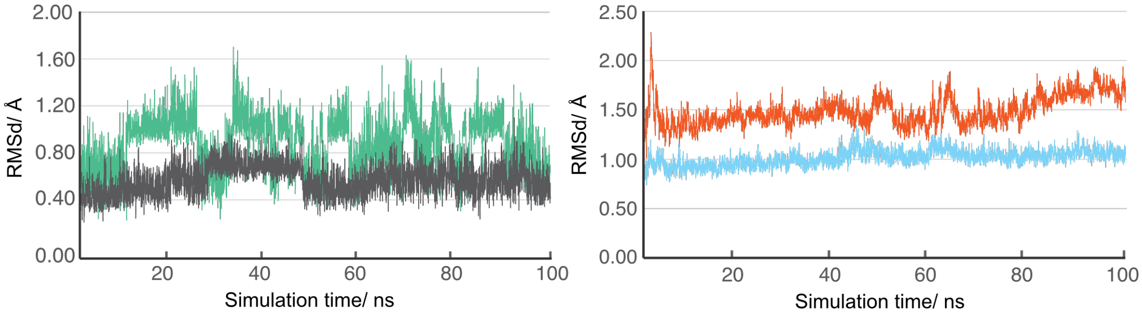

Figure S3- Left) CpdI (dark gray) and L-Tyr (green) RMSd; Right) RMSd of all the backbone (red) and RMSd of the backbone excluding the N-terminal and solvent-exposed loop (light blue).

Table S3- Single-point energies at the B3LYP/6-311+G(2d.2p):ff14SB level of theory for all stationary points along with the respective thermal correction to Gibbs free energy at 298.15K

	$\Delta H_{QM/MM}$/ kcal·mol^{-1}	$\Delta G_{298.15K\ corr.}$/ kcal·mol^{-1}	ΔG/ kcal·mol^{-1}
$REACT_d$	0.00	0.00	0.00
$TS1_d$	-1.14	-1.85	0.71
$INT1_d$	-10.36	-0.57	-9.79
$INT1_d$	-7.43	0.00	-7.43
$TS2_d$	10.63	2.33	8.47
$PROD_d$	-24.17	3.87	-28.03
$REACT_q$	0.00	0.00	0.00
$TS1_q$	-2.27	-3.96	1.69
$INT1_q$	-12.29	-0.57	-11.71
$INT1_q$	-10.53	0.00	-10.53
$TS2_q$	7.90	3.59	4.31
$PROD_q$	-37.98	1.85	-39.83

AMBER compatible force field parameters for Cpd I

MASS

fe	55.85
oa	16.00

BOND

fe-nc	114.00	2.029
fe-nd	114.00	2.029
fe-SH	39.00	2.565
cg-ha	341.50	1.089
fe-oa	572.00	1.639

ANGLE

nc-fe-oa	65.00	92.41
nd-fe-oa	65.00	92.41
SH-fe-oa	0.00	174.09
nc-fe-nd	239.00	89.90
fe-nc-cc	146.00	126.65
fe-nd-cd	146.00	126.65
nc-fe-nc	0.00	174.73
nd-fe-nd	0.00	175.64
SH-fe-nc	48.00	87.60
SH-fe-nd	48.00	87.60
CT-SH-fe	39.00	105.89
cc-cc-cg	65.60	124.54
cd-cd-cg	65.60	124.54
nc-cc-cg	68.50	125.04
nd-cd-cg	68.50	125.04
ha-cc-ha	38.00	117.65
ha-cd-ha	38.00	117.65
cc-cg-ha	46.60	116.97
cd-cg-ha	46.60	116.97
cd-cg-cc	63.80	126.06

DIHEDRAL

cd-nd-fe-oa	1	0.00	180.00	2.00
cc-nc-fe-oa	1	0.00	180.00	2.00
CT-SH-fe-nc	1	0.00	180.00	2.00
CT-SH-fe-nd	1	0.00	180.00	2.00
CT-SH-fe-oa	1	0.00	180.00	2.00
cc-cc-c3-c3	3	0.00	180.00	2.00
cc-cc-c3-hc	3	0.00	180.00	2.00
cd-cd-c3-c3	3	0.00	180.00	2.00
cd-cd-c3-hc	3	0.00	180.00	2.00
X-cg-cd-X	4	16.00	180.00	2.00
X-cg-cc-X	4	16.00	180.00	2.00
cg-cc-nc-fe	1	0.00	180.00	2.00
cg-cd-nd-fe	1	0.00	180.00	2.00
cc-cc-nc-fe	1	0.00	180.00	2.00
cd-cd-nd-fe	1	0.00	180.00	2.00

```
nd-fe-nc-cc       1     0.00   180.00   2.00
nc-fe-nd-cd       1     0.00   180.00   2.00
cc-nc-fe-nc       1     0.00   180.00   2.00
cd-nd-fe-nd       1     0.00   180.00   2.00
cc-nc-fe-SH       1     0.00   180.00   2.00
cd-nd-fe-sh       1     0.00   180.00   2.00

IMPROPER

NONBON
fe             1.30     0.01
oa             1.66     0.21
```

Atomic coordinates and charges for Cys439 provided in the TRIPOS MOL2 format

```
@<TRIPOS>MOLECULE
CYP-CPDI
 10     9     1          0     0
SMALL
USER_CHARGES

@<TRIPOS>ATOM
  1  N      -2.63      -1.35   2.78  N     1  CYP    -0.416
  2  H      -2.83      -2.09   3.45  H     1  CYP     0.272
  3  CA     -1.29      -1.45   2.22  CT    1  CYP     0.073
  4  HA     -1.36      -1.62   1.15  H1    1  CYP    -0.010
  5  CB     -0.48      -0.13   2.42  CT    1  CYP     0.002
  6  HB2    -0.40       0.07   3.50  H1    1  CYP     0.051
  7  HB3    -1.08       0.66   1.98  H1    1  CYP     0.051
  8  SG      1.23      -0.08   1.71  SH    1  CYP    -0.438
  9  C      -0.67      -2.70   2.88  C     1  CYP     0.597
 10  O      -1.28      -3.36   3.73  O     1  CYP    -0.568

@<TRIPOS>BOND
  1     1     3          1
  2     1     2          1
  3     3     4          1
  4     3     5          1
  5     3     9          1
  6     5     6          1
  7     5     7          1
  8     5     8          1
  9     9    10          2

@<TRIPOS>SUBSTRUCTURE
  1  CYP        1  RESIDUE        4  A    CYP      0  ROOT
```

Atomic coordinates and charges for the heme cofactor and oxo ligand provided in the TRIPOS MOL2 format

```
@<TRIPOS>MOLECULE
HEM-CPDI
74      81      1       0       1
SMALL
USER_CHARGES
@<TRIPOS>ATOM
  1  NC     2.87  -0.98  -0.75  nc  1  HEM  -0.033
  2  C1C    4.21  -0.78  -0.53  cc  1  HEM  -0.084
  3  C4C    2.71  -2.31  -1.02  cc  1  HEM  -0.015
  4  C2C    4.93  -2.03  -0.65  cc  1  HEM  -0.003
  5  C3C    4.00  -3.00  -0.94  cc  1  HEM  -0.164
  6  CHD    1.50  -2.92  -1.33  cg  1  HEM   0.047
  7  HHD    1.52  -3.99  -1.50  ha  1  HEM   0.077
  8  C1D    0.28  -2.29  -1.46  cd  1  HEM  -0.126
  9  ND     0.03  -0.96  -1.25  nd  1  HEM   0.069
 10  C4D   -1.30  -0.77  -1.52  cd  1  HEM  -0.034
 11  C3D   -1.94  -2.03  -1.90  cd  1  HEM  -0.033
 12  C2D   -0.95  -2.98  -1.85  cd  1  HEM   0.029
 13  CHA   -1.96   0.45  -1.42  cg  1  HEM  -0.012
 14  HHA   -3.02   0.46  -1.64  ha  1  HEM   0.192
 15  C1A   -1.38   1.65  -1.01  cc  1  HEM  -0.037
 16  C2A   -2.10   2.91  -0.89  cc  1  HEM  -0.063
 17  C4A    0.08   3.13  -0.33  cc  1  HEM  -0.054
 18  NA    -0.07   1.81  -0.66  nc  1  HEM  -0.002
 19  C3A   -1.18   3.83  -0.46  cc  1  HEM   0.010
 20  CHB    1.27   3.74   0.04  cg  1  HEM   0.021
 21  HHB    1.22   4.79   0.29  ha  1  HEM   0.100
 22  C1B    2.53   3.14   0.09  cd  1  HEM  -0.116
 23  C2B    3.75   3.83   0.43  cd  1  HEM   0.081
 24  NB     2.78   1.82  -0.18  nd  1  HEM   0.034
 25  C4B    4.12   1.65  -0.05  cd  1  HEM  -0.087
 26  CHC    4.79   0.44  -0.21  cg  1  HEM   0.057
 27  HHC    5.86   0.44  -0.03  ha  1  HEM   0.074
 28  FE     1.41   0.44  -0.80  fe  1  HEM   0.262
 29  C3B    4.76   2.90   0.34  cd  1  HEM  -0.024
 30  CAB    6.19   3.06   0.54  cc  1  HEM  -0.096
 31  HAB    6.82   2.33   0.03  ha  1  HEM   0.117
 32  CBB    6.84   4.00   1.27  cd  1  HEM  -0.397
 33  HBB1   6.31   4.74   1.85  ha  1  HEM   0.141
 34  HBB2   7.92   4.01   1.31  ha  1  HEM   0.141
 35  CAC    4.18  -4.42  -1.19  cd  1  HEM   0.092
 36  HAC    3.40  -4.89  -1.79  ha  1  HEM   0.077
 37  CBC    5.18  -5.21  -0.76  cc  1  HEM  -0.472
 38  HBC1   5.98  -4.86  -0.12  ha  1  HEM   0.152
 39  HBC2   5.21  -6.27  -1.03  ha  1  HEM   0.152
 40  CMB    3.86   5.29   0.74  c3  1  HEM  -0.126
 41  HMB1   3.80   5.49   1.82  hc  1  HEM   0.040
 42  HMB2   4.83   5.69   0.40  hc  1  HEM   0.040
 43  HMB3   3.07   5.87   0.26  hc  1  HEM   0.040
 44  CMC    6.42  -2.18  -0.51  c3  1  HEM   0.035
```

45	HMC1	6.72	-2.44	0.52	hc	1	HEM	0.005
46	HMC2	6.79	-2.98	-1.16	hc	1	HEM	0.005
47	HMC3	6.95	-1.26	-0.78	hc	1	HEM	0.005
48	CMD	-1.06	-4.45	-2.13	c3	1	HEM	-0.212
49	HMD1	-0.33	-4.77	-2.89	hc	1	HEM	0.065
50	HMD2	-0.87	-5.05	-1.23	hc	1	HEM	0.065
51	HMD3	-2.06	-4.70	-2.49	hc	1	HEM	0.065
52	CMA	-1.39	5.29	-0.17	c3	1	HEM	-0.075
53	HMA1	-1.30	5.51	0.90	hc	1	HEM	0.031
54	HMA2	-0.66	5.92	-0.69	hc	1	HEM	0.031
55	HMA3	-2.39	5.61	-0.49	hc	1	HEM	0.031
56	CAA	-3.55	3.17	-1.19	c3	1	HEM	-0.012
57	HAA1	-3.66	4.13	-1.71	hc	1	HEM	0.025
58	HAA2	-3.95	2.42	-1.88	hc	1	HEM	0.025
59	CAD	-3.40	-2.25	-2.18	c3	1	HEM	-0.066
60	HAD1	-3.82	-1.39	-2.72	hc	1	HEM	0.036
61	HAD2	-3.53	-3.10	-2.86	hc	1	HEM	0.036
62	CBA	-4.44	3.19	0.07	c3	1	HEM	-0.030
63	HBA1	-4.05	3.90	0.81	h1	1	HEM	-0.002
64	HBA2	-4.43	2.21	0.56	h1	1	HEM	-0.002
65	CBD	-4.22	-2.48	-0.90	c3	1	HEM	-0.002
66	HBD1	-4.05	-1.65	-0.21	h1	1	HEM	0.014
67	HBD2	-3.86	-3.39	-0.38	h1	1	HEM	0.014
68	CGD	-5.73	-2.62	-1.12	c	1	HEM	0.575
69	CGA	-5.89	3.58	-0.22	c	1	HEM	0.640
70	O1A	-6.68	3.74	0.76	o	1	HEM	-0.651
71	O1D	-6.46	-2.63	-0.09	o	1	HEM	-0.655
72	O2A	-6.30	3.77	-1.39	o	1	HEM	-0.651
73	O2D	-6.19	-2.73	-2.28	o	1	HEM	-0.655
74	O1	1.69	0.78	-2.37	oa	1	HEM	-0.373

@<TRIPOS>BOND

1	1	2	1
2	1	3	1
3	1	28	1
4	2	4	1
5	2	26	1
6	3	5	1
7	3	6	1
8	4	5	1
9	4	44	1
10	5	35	1
11	6	7	1
12	6	8	1
13	8	9	1
14	8	12	1
15	9	10	1
16	9	28	1
17	10	11	1
18	10	13	1
19	11	12	1
20	11	59	1
21	12	48	1
22	13	14	1

23	13	15	1
24	15	16	1
25	15	18	1
26	16	19	1
27	16	56	1
28	17	18	1
29	17	19	1
30	17	20	1
31	18	28	1
32	19	52	1
33	20	21	1
34	20	22	1
35	22	23	1
36	22	24	1
37	23	29	1
38	23	40	1
39	24	25	1
40	24	28	1
41	25	26	1
42	25	29	1
43	26	27	1
44	28	74	1
45	29	30	1
46	30	31	1
47	30	32	1
48	32	33	1
49	32	34	1
50	35	36	1
51	35	37	1
52	37	38	1
53	37	39	1
54	40	41	1
55	40	42	1
56	40	43	1
57	44	45	1
58	44	46	1
59	44	47	1
60	48	49	1
61	48	50	1
62	48	51	1
63	52	53	1
64	52	54	1
65	52	55	1
66	56	57	1
67	56	58	1
68	56	62	1
69	59	60	1
70	59	61	1
71	59	65	1
72	62	63	1
73	62	64	1
74	62	69	1
75	65	66	1

```
76    65    67    1
77    65    68    1
78    68    71    1
79    68    73    1
80    69    70    1
81    69    72    1
@<TRIPOS>SUBSTRUCTURE
  1  HEM        1  ****        0  ****    ****
```
